# Understanding the Impact of Belzutifan on Treatment Strategies for Patients with VHL

**DOI:** 10.15586/jkcvhl.v9i3.245

**Published:** 2022-09-28

**Authors:** Aileen Arevalo, Neal Patel, Peter Muraki, Shinji Ohtake, Gennady Bratslavsky, Chandra Clark, Joshua Mann, Othon Iliopoulos, Eric Jonasch, Ramaprasad Srinivasan, Brian Shuch

**Affiliations:** 1 David Geffen School of Medicine, University of California, Los Angeles, CA, USA;; 2 Weill Cornell Medicine, New York NY, USA;; 3 Department of Urology, Institute of Urologic Oncology, University of California, Los Angeles, CA, USA;; 4 Urology Department, SUNY Upstate Medical University, Syracuse, NY, USA;; 5 VHL Alliance, Boston, MA, USA;; 6 Center for Cancer Research, Massachusetts General Hospital Cancer Center, Harvard Medical School, Boston, MA, USA;; 7 Department of Genitourinary Medical Oncology, MD Anderson Cancer Center, Houston, TX, USA;; 8 Urologic Oncology Branch, Center for Cancer Research, National Cancer Institute, Bethesda, MD, USA

**Keywords:** VHL, kidney cancer, systemic therapy

## Abstract

Belzutifan was recently approved for the management of Von Hippel–Lindau disease (VHL). Given the morbidity of recurrent treatment, systemic therapy to reduce or eliminate the need for surgery has been long-awaited. Herein, we sought to gain insight about future utilization by surveying VHL kidney cancer experts in the United States. A survey developed by members of the VHL Alliance (VHLA) Clinical Advisory Council was distributed to kidney cancer providers at VHLA and National Comprehensive Cancer Network (NCCN) centers. Surveys were administered on a secure web-based platform. A total of 60 respondents from 29 institutions participated. Urologists (50%) and medical oncologists (43%) represented the majority of participants. The majority (98%) of respondents anticipated that belzutifan’s approval would significantly change the current treatment landscape. Most reported that therapy should be continuous (76%). There was a difference in willingness to prescribe belzutifan by specialty (38% of urologists vs 91% of medical oncologists (P = 0.02)). In individuals with renal tumors <3 cm, 36% would still recommend surveillance, while 36% would initiate belzutifan to prevent growth. In those with multifocal renal lesions and growth of a solitary tumor on belzutifan, 50% would proceed with only treatment of that site. In conclusion, VHL kidney cancer specialists anticipate a paradigm shift with the approval of belzutifan. Provider roles may change with movement away from surgical management. Opinions on treatment indications, such as when to initiate therapy and how to best salvage, vary widely and therefore collaborative efforts among experts may assist in the development of new clinical guidelines.

## Introduction

Von Hippel–Lindau disease (VHL) is an inherited cancer syndrome characterized by the loss of the *VHL* gene, which is a classic tumor suppressor gene. Patients have a predisposition to a wide range of malignant and benign tumors, including central nervous system (CNS) hemangioblastomas, retinal hemangiomas, clear cell renal cell carcinomas (ccRCC), pancreatic neuroendocrine tumors, adrenal pheochromocytomas or paragangliomas, and epididymal and broad ligament cystadenomas (1). Most patients develop multiple disease manifestations starting early in their life and as a result undergo frequent surgical management (2). While ccRCC used to have the greatest mortality due to risk of metastatic progression, modern surgical management has significantly improved survival (3). Currently, median life expectancy extends into the seventh decade with benign CNS hemangioblastomas the leading cause of death (4, 5).

In order to prevent metastatic dissemination, kidney tumors are commonly treated with nephron-sparing approaches, such as partial nephrectomy or ablation (6). Although active surveillance is frequently employed to reduce the morbidity of repeat surgery, once tumors grow larger than 3 cm they have a higher propensity to metastasize, and treatment is generally recommended (7). Unfortunately, despite effective local control with partial nephrectomy, *de novo* recurrences are common, and there are limited additional treatment options except for highly complex reoperative surgery, which carries a high risk of morbidity and risk of nephrectomy (8, 9). Several systemic therapy trials aimed at reducing the need for surgery have been conducted, but none have successfully been integrated into routine clinical care.

Recently, a long-awaited medical treatment option for localized tumors in VHL patients was approved by the Federal Drug Administration (FDA) based on a single arm phase 2 clinical trials. In this study, patients with RCC associated with VHL disease who did not require immediate surgery were given oral belzutifan, which is a small molecule inhibitor of selective hypoxia inducible factor-2 alpha (HIF-2α). A total of 61 patients were enrolled in the trial with a median follow-up of 21.8 months. Overall, 49% of patients had an objective response for renal lesions to belzutifan, and 49% of patients had stable disease. Additionally, efficacy was seen with extra-renal manifestations as 30 and 91% of patients had responses with CNS and pancreatic neuroendocrine tumors, respectively (10). Therapy was very well tolerated with anemia being the most common side effect, and only one patient discontinued therapy due to toxicity. Although these finding have been met with great excitement in the VHL community and the use of belzutifan has now been incorporated into the most recent National Comprehensive Cancer Network (NCCN) guidelines with very broad indications balancing the benefit with long-term usage, toxicity, fertility risks, and the potential development of resistance must be considered. Due to the management complexity of VHL patients and lack of clear guidelines on when to initiate therapy, we sought to survey VHL kidney cancer experts across the United States to gain insight into future utilization.

## Methods

### Expert panel

A multidisciplinary group of VHL kidney cancer experts (urologists, medical oncologists, and patient advocates) was queried to provide perspectives on the use of belzutifan in patients with VHL. Several broad themes emerged regarding the use of belzutifan: (i) indications for kidney specific manifestations, (ii) indications for extra-renal manifestations, (iii) duration of use, and (iv) indications for local treatment on therapy.

### Survey development and administration

An initial list of potential indications for belzutifan use was derived from specific clinical management scenarios that the expert panel had encountered and anticipated being a future challenge. Survey questions were subsequently developed and distributed to an expert panel involving several members from the VHL Alliance (VHLA) Clinical Advisory Council for review and modification. The survey was then built using Qualtrics online software (Seattle, Washington) to capture anonymous web-based responses. The survey was conducted from August to September, 2021, prior to the approval of belzutifan in the United States. Question formats included multiple choice and free text fields. Only practitioners involved in the management of VHL kidney cancer patients were the intended demographic for the survey. Main contacts at VHLA care centers and NCCN centers in the United States were approached to distribute the survey among kidney cancer specialists at their institutions. Multiple clinicians from each institution could respond in order to capture input across specialties. Repeat requests to increase survey response were sent after 2 weeks, with the survey closed after 1 month.

### Data analysis

Stored data from the Qualtrics online platform was obtained, and GraphPad Software v9.0 (San Diego, California) was used for statistical analysis. The surveys were sorted by respondents’ medical specialty, and descriptive statistics were performed to evaluate differences between groups.

## Results

There were 60 survey respondents who were geographically distributed across the United States. Respondents represented 29 different institutions that were VHLA care centers and/or had NCCN designation. The most common medical specialty of respondents was urology (50%), followed by medical oncology (43%), and other (7%) including nephrology or medical genetics. Most providers, 54%, reported seeing <5 VHL patients per year, and 12% saw >20 per year. The respondents reported that the majority of VHL patients (73%) received multidisciplinary input for the management of VHL-associated tumors. However, in terms of kidney cancer management, 68% of participants reported that urologists primarily determined the management.

Most participants (91%) were familiar with the drug belzutifan and the recent clinical trial (NCT04195750) leading to its approval. Participants believed that if approval was received for belzutifan with broad indications, there would be significant treatment implications with expected changes in management paradigms for both renal (98%) and extra-renal (96%) manifestations. Most, 91%, had no concerns managing minor toxicity, and 87% had no concerns with moderate (Grade 3+ toxicity) associated with oral systemic agents such as belzutifan. Almost all (98%) reported as having the capability to measure oxygen saturation in their respective clinics and had no barriers to prescribing erythropoietin-stimulating agents and/or transfusion if needed (85%).

Overall, with the approval of belzutifan, 71% believed that medical oncology will have a larger role in the management of VHL. A significant proportion of urologists (38%) planned to prescribe belzutifan, but less than medical oncologists (91%), P = 0.0262. However, currently, only 7% of the surveyed urologists reported engagement in administration of systemic therapy. With regard to duration of therapy, 76% recommended continuous duration with discontinuation only upon significant toxicity or progression. Most urologists, when compared to medical oncologists (40% vs 10%), were of the opinion that treatment duration should be intermittent (P = 0.033).

For the selection of therapy in VHL patients with kidney tumors, most respondents (87%) preferred to initiate belzutifan in individuals where local treatment would cause significant morbidity, and up to 54% considered starting belzutifan as the primary treatment for lesions meeting the size threshold for intervention. Very few respondents (2%) preferred to start belzutifan prophylactically to prevent the development of tumors. In patients with renal tumors <3 cm and low anticipated morbidity with intervention, 36% preferred to recommend active surveillance and local therapy only if the lesion surpassed the size threshold, 36% would initiate belzutifan to prevent growth, and 28% preferred to recommend active surveillance with consideration of belzutifan only if the lesion surpassed the surgical threshold. In individuals with a renal tumor above 3 cm with low anticipated morbidity, most respondents (86%) still would like to recommend the current approach of local surgical intervention.

With regard to patients who are on therapy, participants were inquired about management strategies for growth while on belzutifan. Local treatment was recommended by a total of 86% of respondents if there were any symptomatic signs of progression, 79% if the lesion reached a predefined threshold of 3 cm, 67% if the tumor exhibited a rapid growth rate, and 26% if there was any lack of significant shrinkage. For VHL patients with multifocal kidney cancer who had growth of a solitary lesion, opinions varied widely by specialty. Overall, 50% of respondents recommended only isolated surgical treatment of that particular lesion with continuation of therapy to treat the other renal tumors. However, urologists are more likely to surgically resect all tumors and not resume belzutifan (60%) compared to medical oncologists who would recommend isolated treatment and continuation of therapy (90%), P < 0.001.

For those with extra renal manifestations, respondents were questioned on treatment indications for belzutifan. The majority (56%) preferred to use belzutifan if local tumor treatment would cause significant morbidity, while 51% preferred belzutifan to shrink tumors that typically required local intervention. However, 44% of respondents stated that they would defer this management decision to the appropriate specialist who manages that given organ site.

## Discussion

In August 2021 after our survey was completed, the FDA approved belzutifan for VHL-associated CNS, pancreas, and kidney tumors that require treatment but not immediate surgery. The landmark approval represents a long-awaited breakthrough for a challenging hereditary cancer syndrome that frequently requires recurrent and morbid surgical management. However, without specific treatment guidelines, there are many unanswered questions regarding its use in routine clinical management. Similarly, as the trial was primarily focused on kidney cancer, the use in pancreas and CNS disease (currently approved) and in retinal and adrenal (unapproved tumor types) disease will likely need to evolve with real world experience given the lack of data from prior systemic therapy options.

Various systemic therapy agents have been used to treat VHL disease, but due to poor efficacy, or poor tolerability, have not been approved. Tyrosine kinase inhibitors such as pazopanib, vandetinib, and sunitinib target broadly downstream in the VHL pathway, which leads to significant off-target toxicity and discontinuation (11–13). HIF-2α has been considered the ideal target, as it is upregulated with loss of the *VHL* gene and is thought to be the main oncogenic driver of both malignant and benign tumors (14, 15). Although prior attempts at drugging this key transcription factor have been largely unsuccessful (16), belzutifan recently demonstrated high efficacy in treating VHL-associated tumors with low toxicity. For renal lesions, the objective response rate was 49% with no patients having progression of disease, and at the time of study publication nearly 90% of individuals remained on therapy (10).

In this study, we assessed the current perspectives on belzutifan for the management of VHL-associated tumors among experts at designated VHL and cancer centers. As expected, 98% of respondents believed that the approval would lead to a paradigm shift in the management VHL-associated kidney cancer. ccRCC is often multifocal, and surgery may be required for the removal of dozens of lesions. Additionally, the high rate of *de novo* recurrence necessitates recurrent partial nephrectomy, which can carry an increased risk of renal insufficiency and potentially morbid urine leaks (8, 17, 18). As a result, there has been a long-standing need for an effective, less invasive therapy. Thus, it is not surprising that there appears to be general consensus that the systemic therapy will have an immediate impact on the care of VHL kidney cancer, and the majority of respondents (87%) plan to use it in individuals with high risk of morbidity with local therapy. However, with a slow rate of growth of most renal lesions, treatment can often be delayed or avoided using active surveillance, leaving the ideal timing of administrating belzutifan unknown.

While there is a common belief that the practice patterns will change, the results of our survey show wide discrepancy in how providers anticipate integrating belzutifan into future treatment paradigms. For individuals with a solid renal tumor that would be a candidate for active surveillance, 43% would consider initiation of belzutifan. For those lesions that would have classically met the threshold for RCC surgery, 54% of providers would recommend initiation of belzutifan instead of surgery. While both are plausible in appropriately selected patients, further data regarding the optimal time to initiate therapy and the long-term toxicity are needed. Despite differences in approaches, there appears to be uniform agreement (99%) that patients should not be given belzutifan prophylactically to prevent the development of new tumors. Giving prophylactically could mean patients are on therapy for an additional number of years. While significant adverse events appear to be rare, little is known about the long-term toxicity and quality of life, as the clinical trial had relatively short follow-up. With regard to treatment duration, only 24% of providers advocate for intermittent therapy which has also been performed with oral therapy in other hereditary cancer syndromes, such as tuberous sclerosis, to minimize drug exposure and toxicity (19).

The morbidity of treatment may play a role in deciding whether to offer local treatment. The recent phase 2 trials show a high rate of tumor stabilization with belzutifan in heavily treated patients (66% with ≥ 4 VHL procedures ≥ 4) who often face a challenging operation and/or loss of kidney function. However, not all operations require a heroic effort, as some tumors (even when multifocal) can be removed with a robotic approach or an outpatient ablation with minimal risk of morbidity. Given the potential concerns of developing metastasis, resistance, and long-term toxicity, expectedly the majority (86%) of respondents would not use belzutifan when a lesion passes the classic threshold and there was low anticipated treatment-related morbidity. Prior to this threshold, most (64%) would not choose to treat such a patient with systemic therapy, as they could be watched and ultimately later undergo a less complicated procedure.

Similarly, wide variations in responses were seen regarding indications for salvage treatment after initiating systemic therapy. It is unclear how therapy impacts disease biology and what growth or lack thereof on treatment means to the risk of dissemination. The majority (83%) would offer treatment if there were signs of local progression or when the tumor reached a maximum size threshold (79%). Once a tumor grows beyond the 3 cm threshold or begins to show signs of invasiveness, the risk of developing metastatic disease increases significantly (7). Given that local therapy can prevent dissemination, it not surprising that majority of experts would recommend intervention, as it is unclear how therapy would impact metastatic potential. Although the utility of metrics such as tumor growth rate and solidification of cystic tumors (often used as indications for treatment when on active surveillance) (20, 21) is less clear for patients on belzutifan, the majority would intervene for rapid growth (67%). Additionally, approximately a quarter of participants would consider local therapy if there was lack of shrinkage or regrowth after a nadir. Although, we anticipate that with the accumulation of real-world data, the use of belzutifan will be more refined, we hope that multidisciplinary evaluations will guide the most appropriate use of therapy.

While we selected only experts from high-volume centers, it was quite surprising that our participants had limited experience with VHL management, as only 46% of participants saw >5 patients a year. Although, specialty participation may have impacted the reported experience with VHL, as 60% of urologists and 27% of medical oncologists saw >5 patients per year, it demonstrates that if medical oncologists fill a larger role, there may be need for significant collaboration. To rapidly aid in integration into clinical care, it may take significant coordinated effort to share experiences with belzutifan across centers to help better accumulate additional data on the clinical utility. Fortunately, current efforts are underway with the initiation of a VHL Tumor Board sponsored by VHLA.

Lastly, with the introduction of a well-tolerated oral agent, the question remains as to who will administer the therapy. There was a clear difference of opinion on who should primarily manage systemic therapy. Unlike tyrosine kinase inhibitors which have a high risk of Grade 3 toxicity, belzutifan is generally well tolerated, and the most common adverse events reported are anemia in 90% of the patients and fatigue in 66% (10). Most providers feel comfortable managing side effects associated with oral systemic therapy, and there appears to be resources for broad use across disciplines with the capacity to measure pulse oximetry in clinics and ability to administer erythropoietin-stimulating agents, or blood transfusion, if needed. Only 9% of medical oncologists believed that urologists should administer therapy when compared to 38% of urologists. Given that most of the care is being driven by urology, even in specialized centers, there will need to be more efforts to improve education and multidisciplinary care.

There are several limitations of our survey. At the time of the survey, belzutifan had not been approved by the FDA, and thus our questions were based on challenges that would be anticipated with its use. Firstly, further clinical data will be needed to determine the challenges that integrating this systemic therapy into the management of VHL patients will pose. Secondly, our questions were largely based on the current treatment paradigm. The validity of traditional measures, such as the 3 cm intervention threshold, may no longer be valid with patients on belzutifan. Whether or not belzutifan can change the natural history of VHL-associated kidney cancer is unclear and will require further follow-up data. Lastly, although the survey was widely distributed across multiple centers, there was only representation from clinicians who are highly proficient at managing VHL. Additional clinical experience from the community and medical specialties, such as nephrology and interventional radiology that often play a critical role in the multi-disciplinary care of VHL patients, is needed.

## Conclusions

VHL kidney cancer specialists believe that the recent approval of belzutifan will lead to a paradigm shift and provider roles may change, with movement away from local treatment especially in patients with high risk for morbidity with invasive treatment. However, there is no uniform consensus on the best time to initiate therapy with belzutifan or how to best salvage patients who progress. Additional clinical data and experience is needed to help usher in a new era of VHL management.
